# Supplementary figures and images for: Vibrio cholerae’s mysterious Seventh Pandemic island (VSP-II) encodes novel Zur-regulated zinc starvation genes involved in chemotaxis and cell congregation
Source: PLoS Genet. 2021 Jun 21;17(6):e1009624. doi: 10.1371/journal.pgen.1009624 (PMC8248653; doi:10.1371/journal.pgen.1009624)

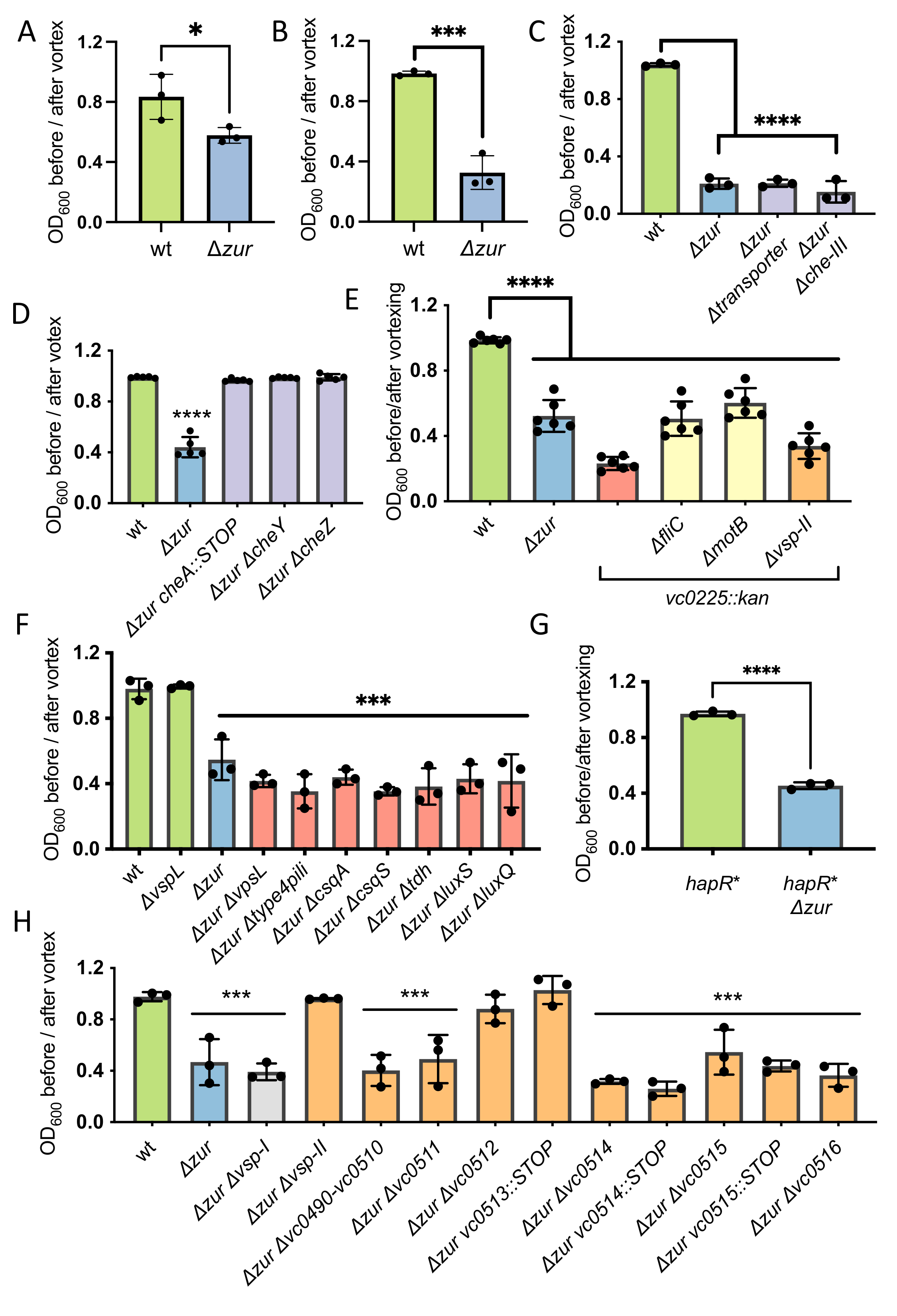

Supplement: S1 Fig — (A-H) All strains were grown overnight in M9 minimal medium plus glucose (0.2%). All cultures were grown shaking (200 rpm), with the exception of static growth tested in panel (A). All cultures were grown in borosilicate glass tubes, with the exception of plastic tubes used in panel (B). Congregation was quantified by measuring the optical density (at 600 nm) of the culture supernatant before and after a brief vortex. The following mutants were tested in a Δzur background: (C) other putative Zur-regulatory targets (ABC-type transporter, Δvca1098-vca1101; che-III cluster, Δvca1090-vca1097), (D) chemotaxis genes (cheA::STOP, ΔcheY, ΔcheZ), (F) biofilm formation genes (ΔvspL), type IV pili (ΔtcpA, ΔmshA, ΔpilA, and Δvc0502), quorum sensing genes (ΔcsqA, ΔcsqS, Δtdh, ΔluxS, or ΔluxQ), (G) N16961 hapRrepaired, (H) the Vibrio Seventh Pandemic (VSP) island -I (Δvc0175-vc0185), and regions of VSP-II (“ΔVSP-II”, Δvc0491-vc0515; Δvc0490-vc0510, Δvc0511, Δvc0512, Δvc0513 or vc0513::STOP, Δvc0514 or vc0514::STOP, Δvc0515 or vc0515::STOP, or Δvc0516). (E) Congregation was also measured in a rough mutant (vc0225::STOP) and a rough mutant harboring deletions for ΔfliC, ΔmotB, or Δvsp-II. Data points represent biological replicates, error bars represent standard deviation, and asterisks denote statistical difference relative to the wild-type strain via (A,B,G) unpaired t-test or (C-F, H) Ordinary one-way ANOVA (****, p < 0.0001; ***, p < 0.001; *, p < 0.05). (TIFF) [file pgen.1009624.s001.tiff]

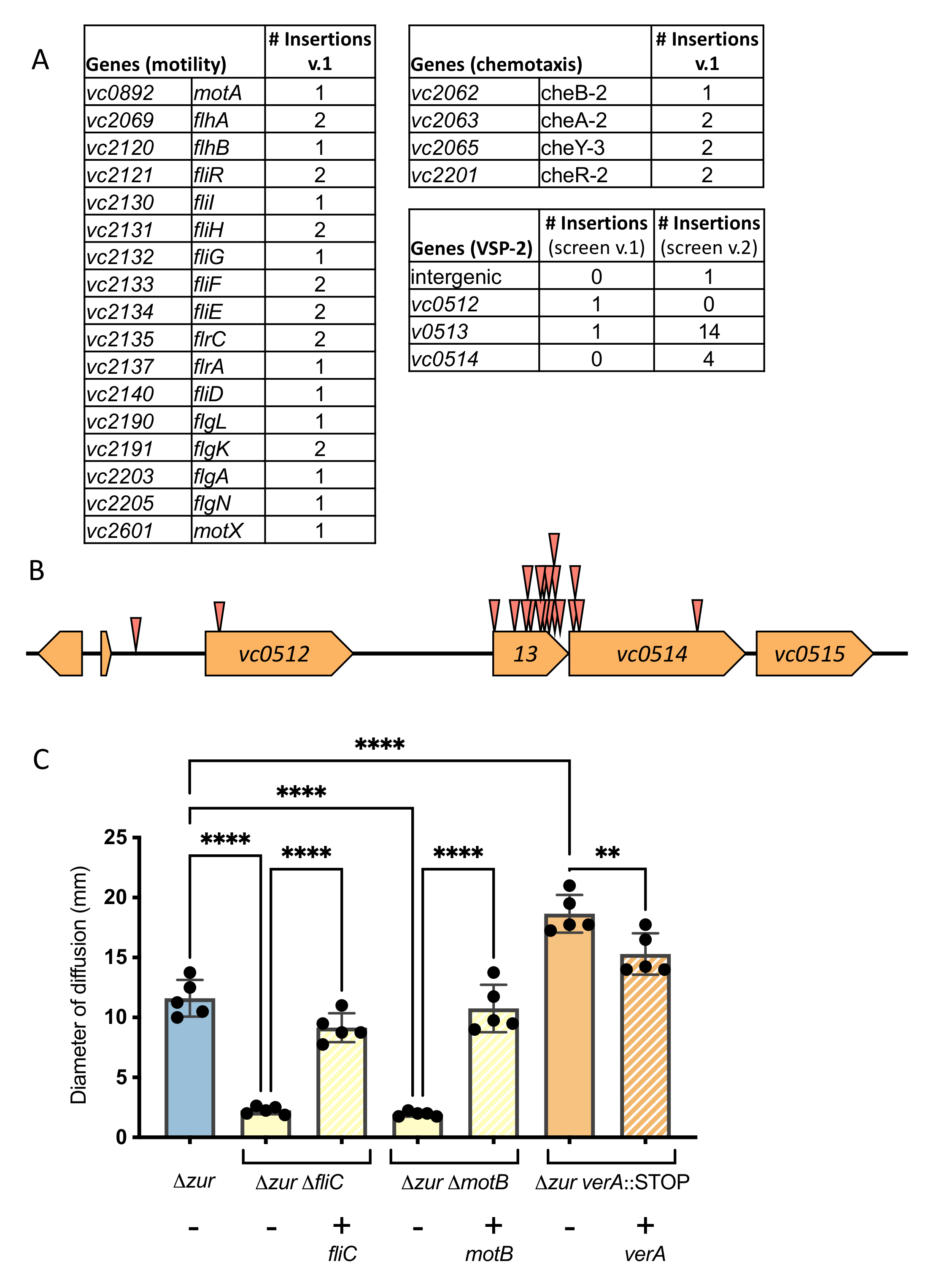

Supplement: S2 Fig — (A) Table indicating the number of transposon insertions within motility, chemotaxis, and VSP-II genes for each of the screens (without pre-selection, v.1; with pre-selection of motile mutants, v.2) described in Fig 2. (B) Approximate location of transposon insertions (triangles) determined by arbitrary PCR [46] and Sanger sequencing are shown. (C) Strains carrying either an empty vector (-) or complementation vector (+) were grown overnight in LB medium with kanamycin. Strains were washed thrice with M9 minimal medium. A sterile toothpick was used to inoculate cells into M9 soft agar (0.3%) containing glucose (0.2%), kanamycin, and inducer (IPTG, 500 μM). The diameter of diffusion (mm) was measured following a 48-hr incubation at 30°C. Raw data points represent biological replicates, error bars represent standard deviation, and asterisks denote statistical difference via Ordinary one-way ANOVA test (****, p < 0.0001; **, p < 0.01). (TIFF) [file pgen.1009624.s002.tiff]

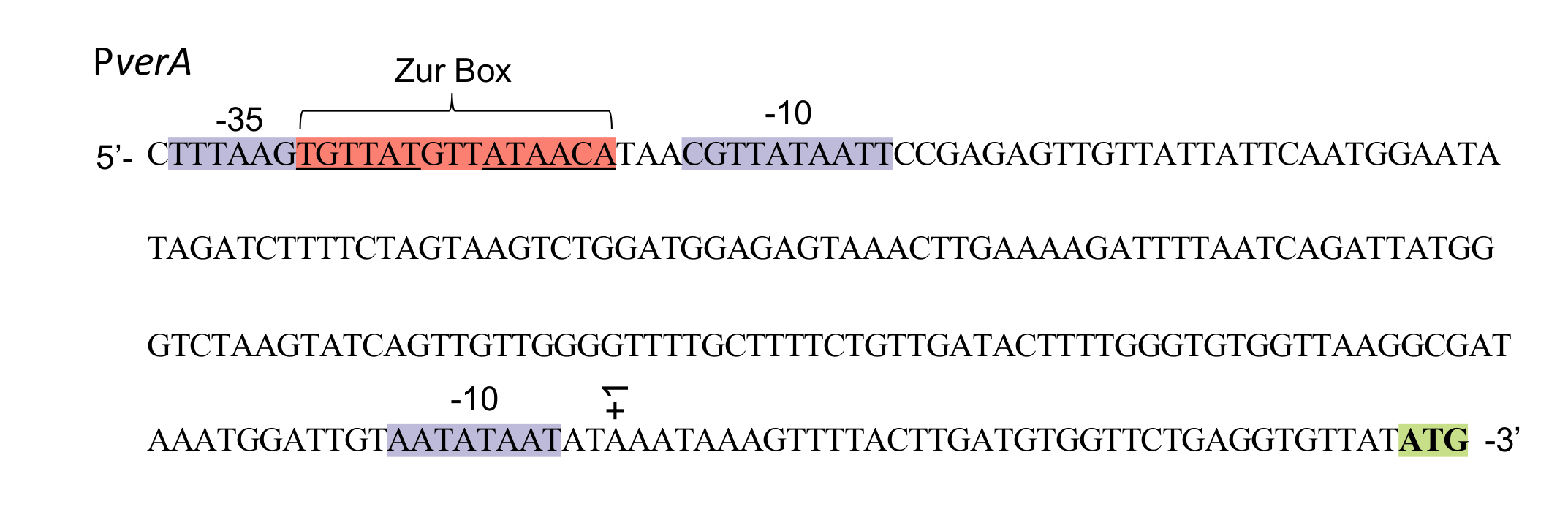

Supplement: S3 Fig — Diagram of the verA promoter region annotated with theh following features: predicted Zur box (red), predicted -10 and -35 regions (purple) [126], suggested start codon (ATG, green). (TIFF) [file pgen.1009624.s003.tiff]

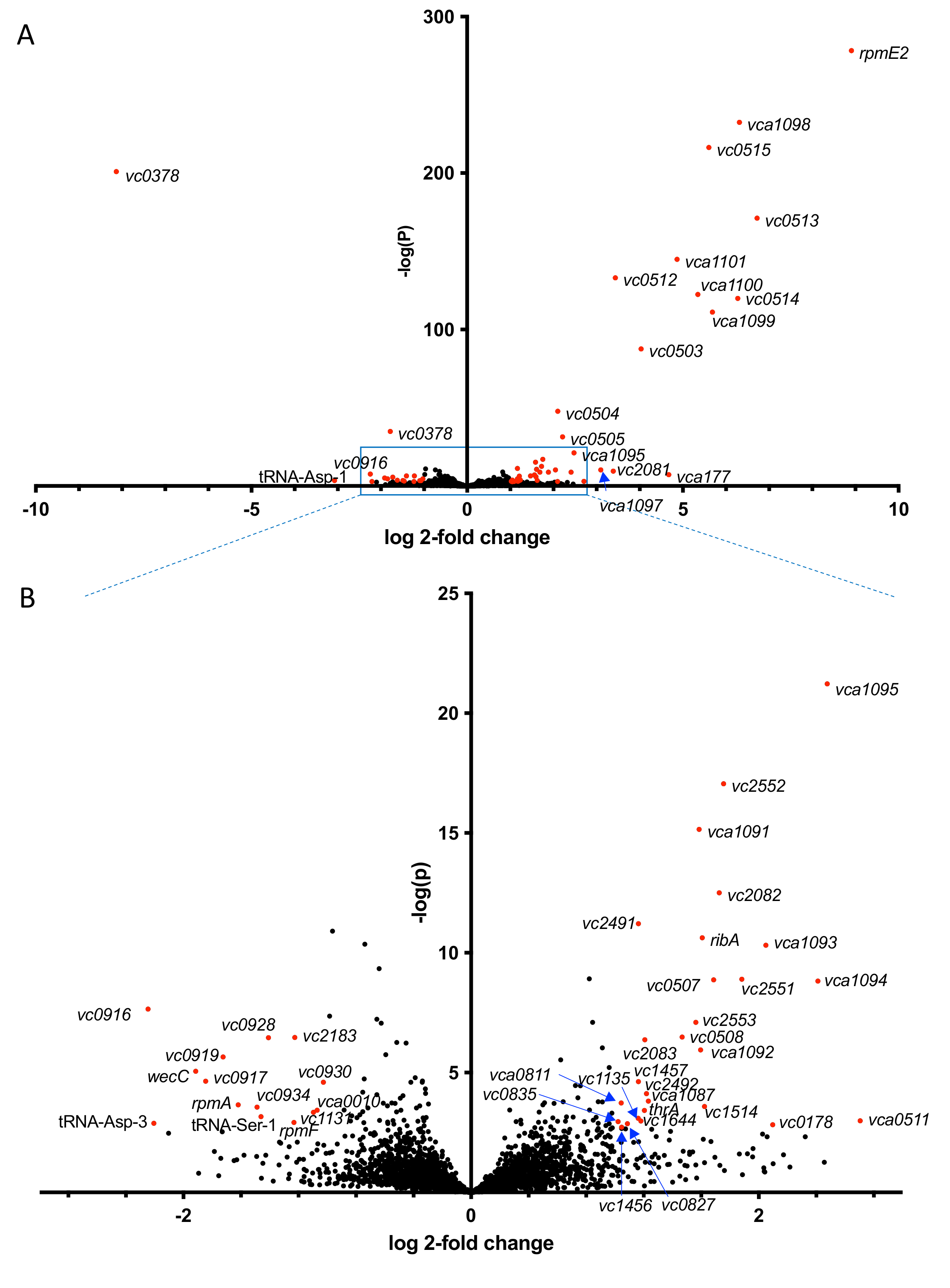

Supplement: S4 Fig — (A-B) Volcano plots showing log 2-fold changes in gene expression in Δzur relative to wild-type; positive values represent up-regulation in Δzur and negative values represent down-regulation in Δzur. The y-axis denotes the negative log inverse of the p-value. Differentially expressed genes (log 2-fold change >1, adjusted p-value < 0.05) are denoted in red and are labeled with gene identifiers. Panel (B) shows the subset of genes within the blue box in Panel (A). (TIFF) [file pgen.1009624.s004.tiff]

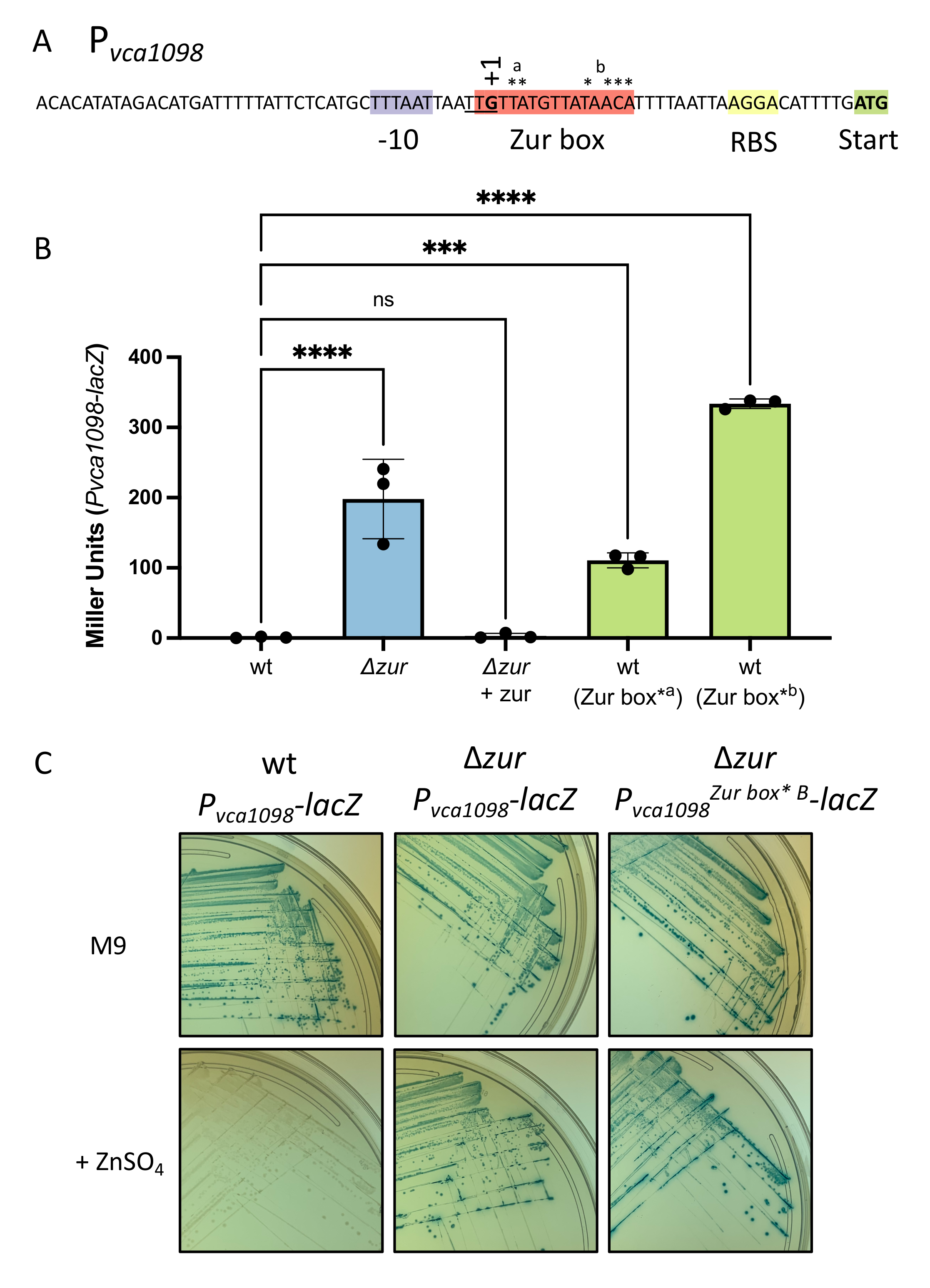

Supplement: S5 Fig — (A) Diagram of the vca1098 promoter region annotated with the following features: predicted Zur box, red; predicted -10 region, purple [126]; transcription start site, +1 (5’-RACE); predicted ribosome binding site (RBS), yellow; proposed start codon (ATG), green. Asterisks indicate Zur box nucleotides (region “a” or “b”) that were altered in the mutant reporters described below. (B) vca1098 promoter lacZ transcriptional reporters (Pvca1098-lacZ, solid bars) or mutated versions (Pvca1098Zur box* a or b-lacZ, striped bars) were inserted into a wild-type or Δzur background harboring a plasmid-borne, IPTG-inducible copy of zur (+) or empty vector control (-). Strains were grown overnight in LB and kanamycin, diluted 1:100 in fresh media containing inducer (IPTG, 400 μM), and grown for 3 hours at 37°C. Promoter activity (in Miller Units) was measured via β-galactosidase assays (See Methods and Materials). (C) Wild-type and Δzur strains carrying Pvca1098-lacZ or mutant derivatives were streaked onto M9 minimal medium agar with glucose (0.2%), X-gal, and with or without added zinc (ZnSO4, 10 μM). Plates were incubated overnight at 30°C and then for an additional day at room temperature. vca1098 promoter activity is signified by a blue colony color. (TIFF) [file pgen.1009624.s005.tiff]

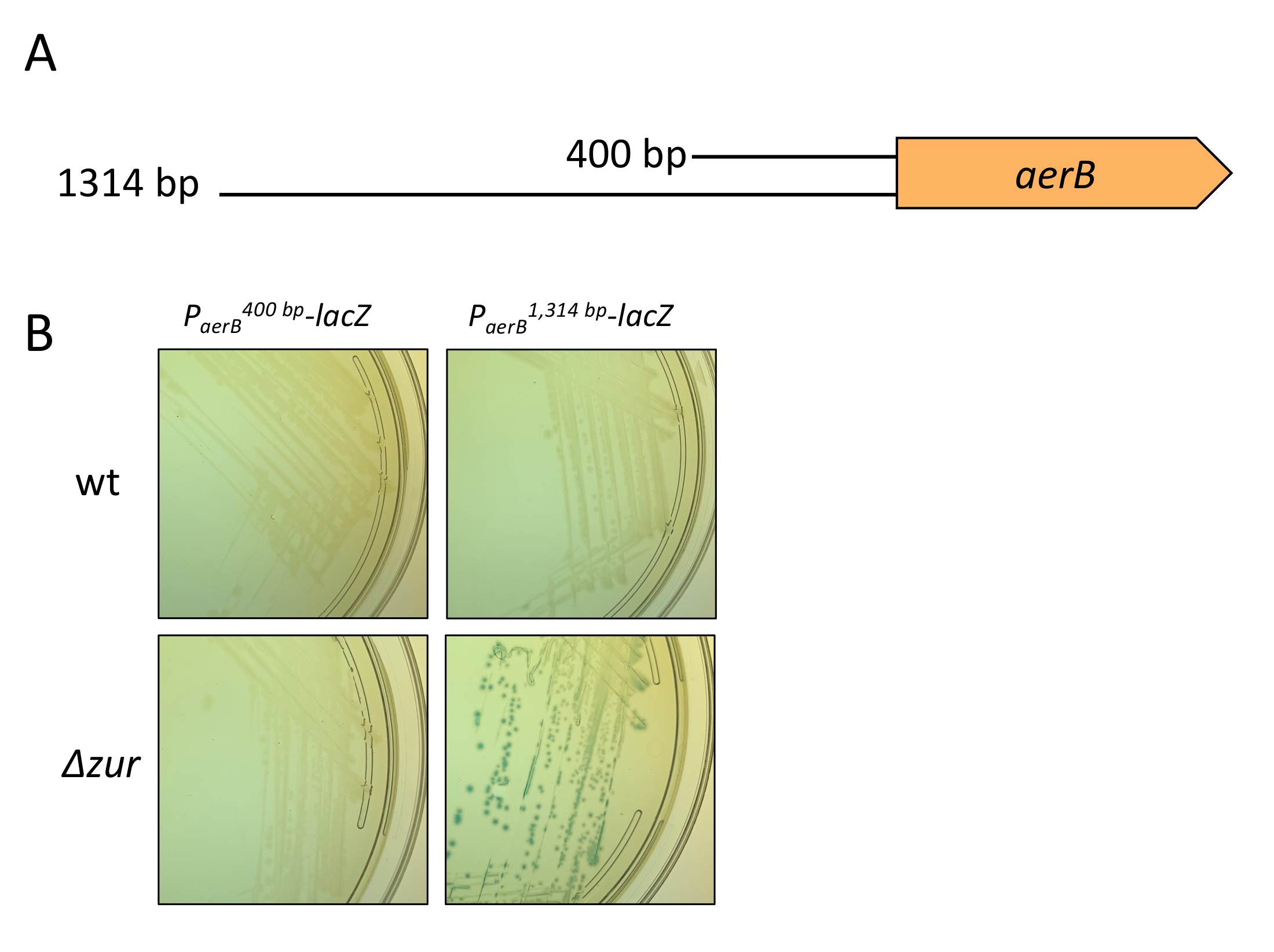

Supplement: S6 Fig — (A) Schematics for two attempted PaerB-lacZ reporters containing either 400 bp or 1,314 bp of the promoter region are shown. (B) The PaerB400 bp-lacZ and PaerB 1,314 bp-lacZ reporters were integrated into a wild-type or Δzur background and were struck onto LB X-gal plates. Plates were incubated overnight at 30°C and then for an additional day at room temperature. PaerB expression is indicated by a blue colony color. (TIFF) [file pgen.1009624.s006.tiff]

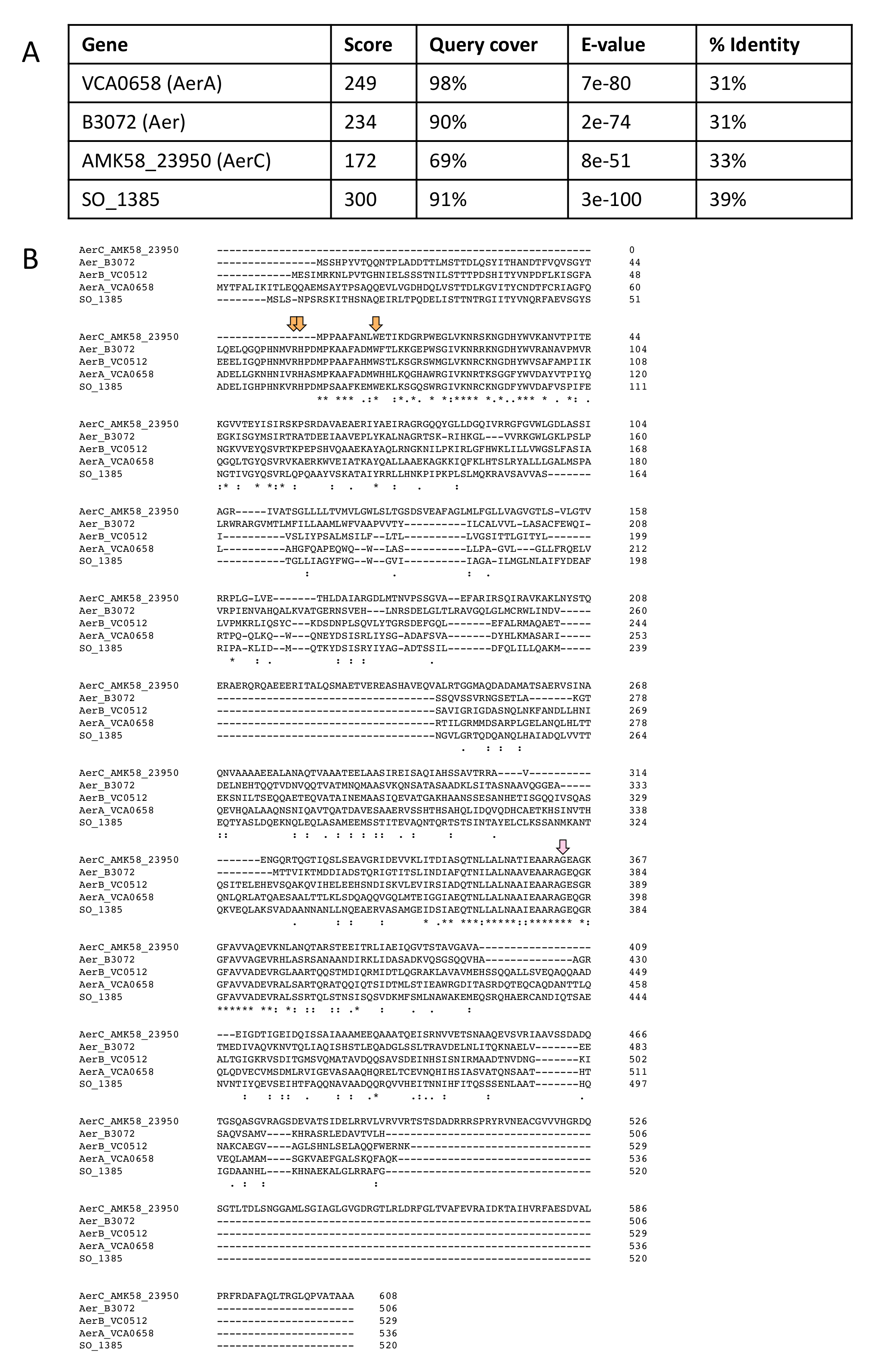

Supplement: S7 Fig — (A) Results of protein BLAST [121] and (B) Clustal Omega alignment [127] of AerB (VC0512) with homologs from V. cholerae (AerA/VCA0658), E. coli (Aer/B3072), A. brasiliensis (AerC/AKM58_23950), and S. oneidensis (SO_1385). Conserved ligand binding and MCP residues targeted for mutation are indicated by orange and pink arrows, respectively. (TIFF) [file pgen.1009624.s007.tiff]

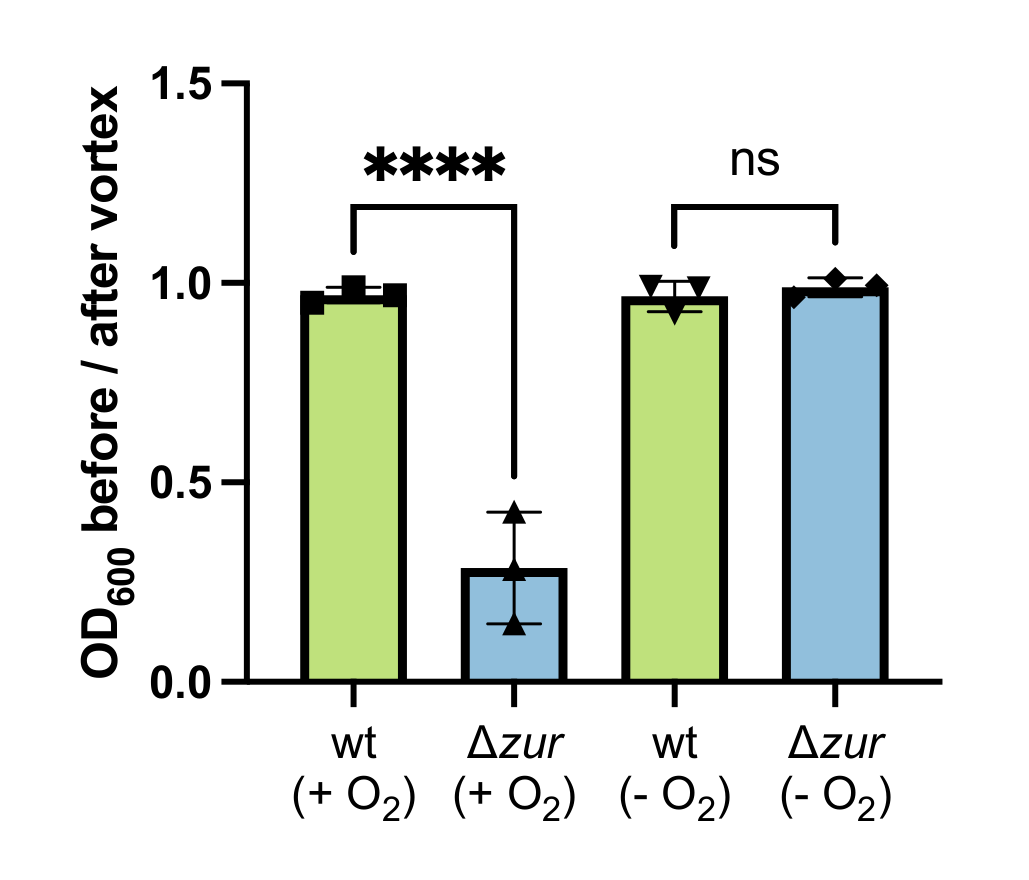

Supplement: S8 Fig — Wild-type and Δzur were grown overnight in 5 mL M9 minimal medium plus glucose (0.5%) fermentatively (without a terminal electron acceptor) and cultured under aerobic (+ O2) or anoxic (- O2) conditions (see Methods for details). Tubes were grown shaking overnight at 30°C and congregation was quantified via spectrophotometry as described previously. All data points represent biological replicates, error bars represent standard deviation, and asterisks denote statistical difference via Ordinary one-way ANOVA test (****, p < 0.0001; n.s., not significant). (TIFF) [file pgen.1009624.s008.tiff]

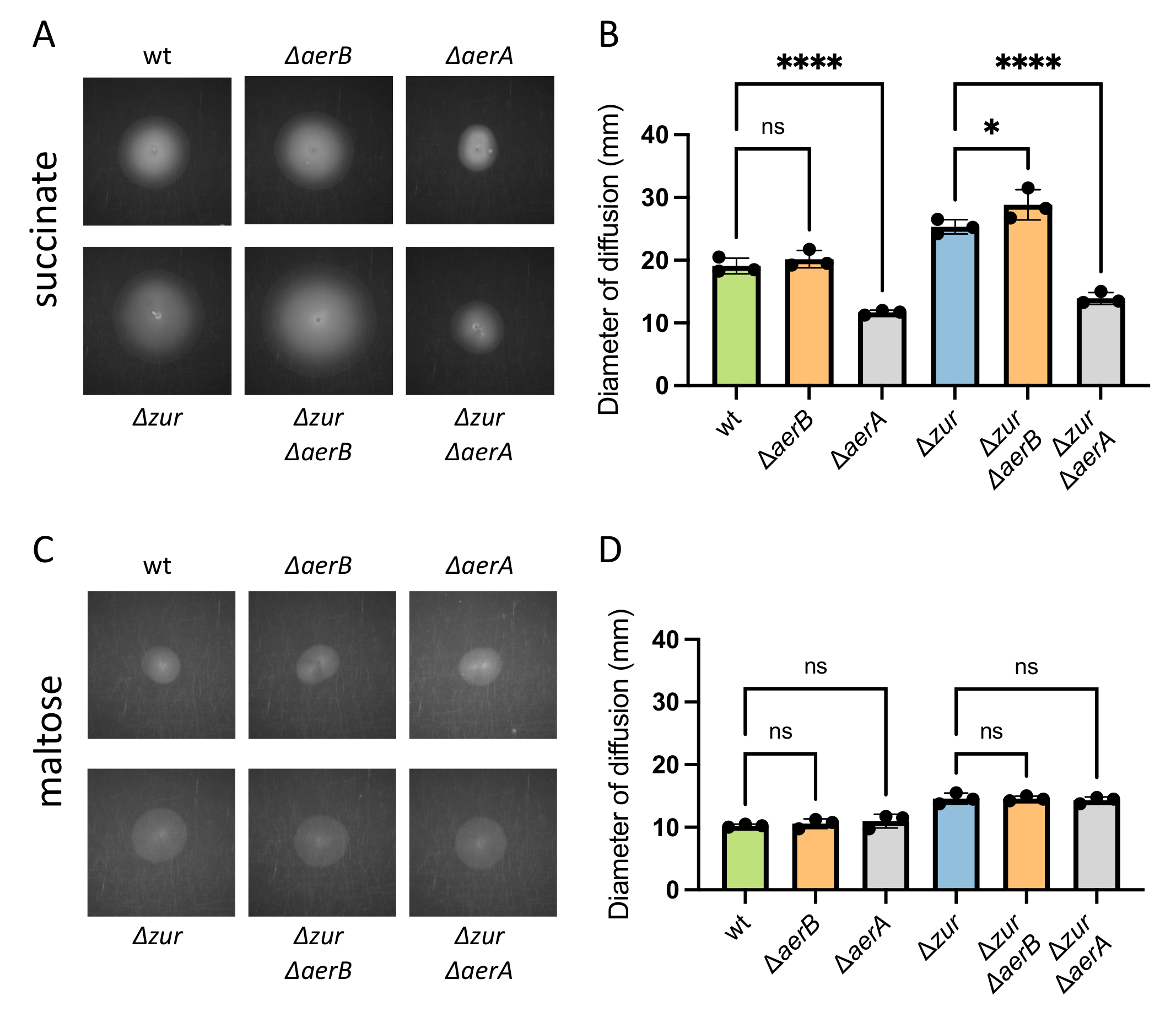

Supplement: S9 Fig — (A-D) The indicated strains were grown overnight in LB medium and washed thrice in M9 minimal medium lacking a carbon source. A sterile toothpick was used to inoculate cells into M9 soft agar (0.3%) with either (A-B) succinate (30 mM) or (C-D) maltose (0.1 mM) as a carbon source. The diameter of diffusion (mm) was measured following a 48-h incubation at 30°C and representative swarms are shown (A,C). Note: Data for Δzur, Δzur ΔaerB, and Δzur ΔaerA are the same as shown in Fig 5 and are shown here for comparison with a wild-type background. All data points represent biological replicates, error bars represent standard deviation, and asterisks denote statistical difference via Ordinary one-way ANOVA test (****, p < 0.0001; ***, p < 0.001, and n.s., not significant). (TIFF) [file pgen.1009624.s009.tiff]

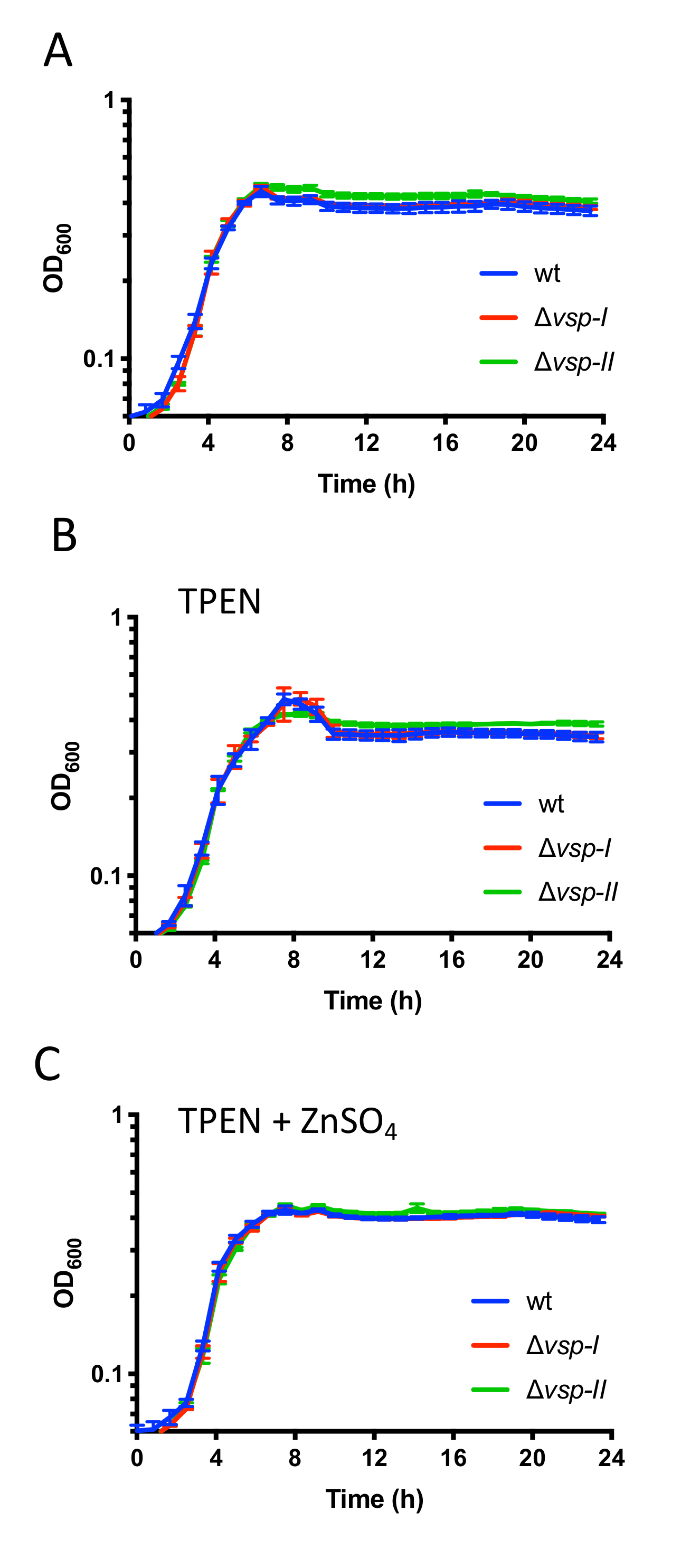

Supplement: S10 Fig — Wild-type, Δvsp-I, and Δvsp-II were grown overnight in M9 minimal medium with glucose (0.2%) at 30°C. Cultures were washed twice and diluted 1:100 into (A) fresh M9 minimal medium glucose (0.2%), (B) plus the zinc-specific chelator TPEN (250 nM), or (C) plus TPEN and exogenous zinc (ZnSO4, 1 μM). Growth at 30°C of each 200-μl culture in a 100-well plate was monitored by optical density at 600 nm (OD600) on a Bioscreen C plate reader (Growth Curves America). (TIFF) [file pgen.1009624.s010.tiff]
