# Supplementary material for: Vibrio cholerae’s mysterious Seventh Pandemic island (VSP-II) encodes novel Zur-regulated zinc starvation genes involved in chemotaxis and cell congregation
Source: PLoS Genet. 2021 Jun 21;17(6):e1009624. doi: 10.1371/journal.pgen.1009624 (PMC8248653; doi:10.1371/journal.pgen.1009624)
Supplement: Attachment — Submitted filename: Murphy_Response to Reviewers.docx [file pgen.1009624.s015.docx]

**Comments to the Authors:
Please note here if the review is uploaded as an attachment.**

Reviewer #1: The manuscript by Murphy et al. identifies a novel role for the second Vibrio pathogenicity island (VSP-II) in the physiology of toxigenic Vibrio cholerae. Specifically, they identified that V. cholerae ∆zur mutants aggregate out of solution when grown aerobically in minimal medium, and then conduct a number of genetic screens which led them identify a regulatory module on VSP-II that was critical for this response. This regulatory module is composed of a transcriptional activator, VerA that induces the expression of a chemotaxis gene, AerB. Using carefully controlled genetic experiments and chemotaxis assays they nicely demonstrate that AerB is sufficient to induce this response and likely promotes a negative chemotactic response to oxygen. The importance of this physiological response during the V. cholerae lifecycle remains unclear, but the authors speculate on this point in the Discussion of the manuscript. Overall, this study was well conducted and very clearly written. The findings represent an important advance to our understanding of V. cholerae biology – especially because it identifies the function of genes associated with one of the genomic islands that distinguish isolates from the current pandemic. I have only minor points for the authors to consider. Namely, to speculate on and or test further the mechanism underlying bacterial aggregation during this response.

We thank the reviewer for their feedback on this work.

Based on the data presented in Fig. 1C it is assumed that the WT grown without ZnSO4 does not experience Zinc starvation. Is this expected due to the efficiency of zinc uptake and the trace amounts of zinc available in the medium? Data in Figure 3 using their PverA reporter construct suggest that this may be the case, but the authors should formally comment on this point earlier in the text.

Yes, the WT does not seem to experience significant zinc starvation in these experiments when grown in M9 minimal medium liquid culture, as evidenced by the data shown in Figure 3C. This suggests that there are sufficient trace amounts of zinc available in in this batch of medium. We have amended the text to specifically comment on this point.

Line 142 – “S1F” not “SF” Corrected.

Figure S1F – does the ∆type IV pili strain really have VC0504 mutated or is it supposed to read VC0502? If the former, an explanation might be warranted since this is not a putative major pilin gene.

This was a typo and has been corrected to read “*vc0502*”.

It is striking that verA was hit so many times in the screen, while aerB, which is sufficient to carry out the phenotype, was only hit once. Can the authors speculate on why this may have occurred? It is not essential to test this experimentally, but do they know if aerB mutants were poorly represented in their input Tn mutant pool?

Yes, the second transposon screen yielded many more insertions to *verA* than *aerB*. We found *verA* mutation (in a *zur* background) caused an increased swarm diameter relative to *zur* (new data in **Figure S2C**), whereas a *zur aerB* mutant has a slight motility defect (**Figure 5**). Since we selected transposon mutants from the outer regions of the swarm plate during the screen, we likely introduced a sampling bias towards *verA* mutants relative to *aerB* mutants. We amended the manuscript to include this point and included these motility data.

Line 160 and 180 – Define the “vc0513-vc0515 promoter region” as “PverA” earlier in the manuscript, since this term is already used. Amended.

Line 163-164 - It is unclear how introduction of a premature stop codon would be more likely to prevent polar effects on downstream genes because Rho-dependent termination could, in fact, still influence the transcription of downstream genes. An in-frame deletion on the other hand would be less likely to suffer from this shortfall. The authors nicely demonstrate that the genes can be complemented in trans, so I am not questioning the results obtained in any way. But I would suggest that they remove this general statement about stop codons unless they can provide additional supporting evidence for it.

We have removed this statement.

Line 213 – should refer to “S1B” Corrected.

Line 241 – refer to Fig. 4B at the end of this sentence. Corrected.

Line 319 – “taxis” not “axis” Corrected.

Line 1100 – vca0685 is an ABC transporter component, not an MCP. Should be vca0658 according to the Methods section. We have amended both the text and the figure.

Figure S7 - The TPEN concentrations used don’t seem to generate zinc limiting conditions for V. cholerae growth since growth kinetics and final growth yield appear unchanged compared to growth when TPEN is omitted. Would higher doses of TPEN reveal a difference in growth? Even if there is not a difference in optical density, could there be a difference in viability that is revealed by quantitative plating? This may fit with the authors hypothesis that this response is required for optimal resistance to redox stress.

TPEN is tricky. We have found that it is very difficult to titrate zinc starvation levels to the point where growth is reduced, but not entirely inhibited. We have used a concentration that is sufficient to induce a transcriptional fusion of *shyB*.

It is striking that aggregation and cell pelleting is the end point phenotype observed if AerB simply promotes a negative chemotactic response to oxygen. If this was simply chemotaxis away from oxygen, I would expect cells to simply swim to the lower part of the culture tube, not actually aggregate out of solution. Is aggregation actually occurring through cell-cell interactions promoting the formation of bacterial clumps? This could be easily assessed by microscopy. If bacteria do form clumps, can the authors speculate on what is promoting cell-cell interactions? Their results nicely demonstrate that the canonical factors thought to mediate cell-cell interactions in V. cholerae are not playing a role here. Since motility seems to play a role in this response could it be tangling of the polar flagellum between cells?

We have added microscopy images of wild-type and ∆*zur* pellets to Figure 1. The presence of many individual cells in the *zur* mutant suggests that there may not be strong-strong interactions holding the pellet together, as any interactions appear to be easily disrupted by pipet transfer or gentle agitation (Movie S1). Beyond oxygen-dependent chemotactic migration, we do not fully understand why *V. cholerae* forms a concentrated pellet at the bottom of the tube.

Since aggregation, in reference to *V. cholerae*, typically implies some type of cell-cell interaction, we have amended the manuscript to refer to this phenomenon as “congregation”, a term which has been used to describe a PAS/chemotaxis-dependent accumulation phenotype observed on *Shewanella* (doi: 10.3389/fmicb.2017.02568).

Is the oxygen-dependent aggregation response reversible? For example, if ∆Zur or AerB overexpressing cells are grown aerobically and then transitioned to anaerobic conditions, do bacterial aggregate dissipate? If AerB is directly sensing oxygen to modulate motility as proposed, I would expect this response to be reversible.

Our anaerobic experiments were performed in anoxic culture tubes. This experiment would require shifting aerobically grown tubes into an anaerobic chamber; since we do not currently have access to an anaerobic chamber due to COVID-19 restrictions, it is difficult to test this hypothesis.

AerA seems to carry out the opposite phenotype to AerB. Do ∆zur ∆aerA mutants aggregate more quickly than ∆zur mutants? This might be expected if the aerotaxis response is sufficient to promote aggregation.

We attempted to express aerA ectopically in a zur mutant to see if it reduced or prevented aggregation. The pellet still formed, suggesting that either aerA cannot reverse aerB-mediated aggregation, or that AerA is not expressed in our strain. Ectopic expression from our aerA construct did not yield any significant differences in motility that have been reported elsewhere (10.1111/j.1574-6968.2007.00931.x), so we suspect that we may not have significant expression. Given the ambiguity of this dataset, we have not included it in the manuscript. We have softened our conclusions in them manuscript and refer to the phenotype observed as oxygen-dependent energy taxis.

It may be worth mentioning in the Discussion that AerB could simply be upstream of additional genes required for this response. The authors nicely demonstrate that ectopic expression of AerB recapitulates the aggregation phenotype even when Zur is intact. In the future, they may want to consider carrying out a genetic selection in the AerB overexpressing strain. Since aerB was poorly hit in their original screen, this modified screen setup may help uncover additional genes that are required for this response.

We found that ectopic *aerB* expression in a mutant lacking the entire VSP-II island generates aggregates, it therefore seems unlikely that other upstream VSP-II genes are required for this response. As described above, we now note in the manuscript aerB is poorly hit in the original screen relative to verA and have included supplemental data to explain why these genes may have been under- or overrepresented in the screen, respectively. We will consider performing this additional screen in follow-up work.

Reviewer #2: I really enjoyed reading this manuscript “Vibrio cholerae’s mysterious Seventh Pandemic island (VSP-II) encodes novel Zur regulated zinc starvation genes involved in chemotaxis and autoaggregation”. In this work, the authors examined an aggregation phenotype in V. cholerae Zur mutants, which mimic a zinc starvation response. They characterize the downstream pathway leading to this novel zinc-starvation response including induction of a transcriptional regulator, VerA, within VSP-II. They further elaborate on the VerA regulon using RNA-Seq to show that it induces expression of its own operon as well as the neighbouring gene, AerB. AerB instigates energy taxis away from oxygen, resulting in the observed aggregation phenotype.
Overall the experiments are well conducted, have appropriate controls and supporting data, and are clearly presented. While the biological role of this phenotype and the in vivo environment where it is relevant remain unknown, the results presented support the conclusions made. These findings provide a significant step forward to the field by describing a novel genetic pathway, phenotype, and energy taxis mechanism in V. cholerae, with potential links to virulence.

We thank the reviewer for their feedback on this work.

1) I am still intrigued by what the biological role of this pathway and phenotype could be. The authors noticed the aggregation phenotype using a zur mutant but did not find a condition that could induce similar phenotype in wild type. Could it be that there is environmental Zn or that Zur responds to other metals?

Although we were only able to observe this phenotype genetically (through zur or znuABC deletion or aerB overexpression), we were able to prevent aggregate formation by manipulating growth conditions (i.e. by adding exogenous zinc to a znuABC mutant, or growing zur under anoxic conditions). It is possible that other environmental cues modulate expression/activity of these genes of interest and we now emphasize this in the discussion.

2) Regarding the aggregation phenotype, the data presented supports swimming away from oxygen, but the modest effect in the succinate swarming assay does not look strong enough to hold the cells together upon vortexing. It seems that there must be something sticking the cells together in an aggregate. You already tested known pili, vps etc., and your Zur RNA-Seq suggests VPS/biofilm are also downregulated. Could any of the other hits in the RNA-Seq of the Zur mutants be possible candidates? Were any of these knocked out to test aggregation?

Beyond the canonical factors already tested, we did not find any additional genes in the RNA-seq that might mediate this phenotype. However, if such aggregation factors are growth medium-dependent, they may not have shown up on our RNA-seq (which was conducted in LB medium). Our transposon screen, which was conducted in M9, did not yield any redundant hits to follow up on.

If there are cell-cell interactions holding these pellets together, they appear to be weak / easily disrupted (images shown in Figure 1). As far as we know, the pellet formation is reliant upon chemotactic migration rather than canonical cell-cell interactions. Since aggregation typically implies cell-cell interactions, we amended the manuscript to describe this phenomenon as “congregation”, a term which has been used to describe a PAS/chemotaxis-dependent accumulation phenotype observed on *Shewanella* (doi: 10.3389/fmicb.2017.02568).

3) The zur mutant was found to aggregates at the bottom of the tube after overnight incubation with shaking. One would think that the oxygen gradient in the shaking tube is not simply from top aerial-liquid interface to tube bottom. How could this bottom aggregation in shaking tubes be driven by AerB-mediated oxygen sensing?

The tubes used for this experiment are ~15 mL capacity and contain 5 mL of volume. We anticipate that even with gentle shaking, there will still be an oxygen gradient present in the cultures. In new data (Fig S1A), we show that aggregation also occurs in zur mutants grown statically at 30 degrees, but we note that it is somewhat less pronounced. It is possible that other factors contribute to formation of these pellets – perhaps the rotational movement of the tubes, accumulation of insoluble material, etc. also influence pellet formation.

4) Maybe it is my personal preference but please do not use the subjective term “mysterious” in the title and text.

If the editor agrees with Reviewer #2, we will remove mysterious from the title. However, it is our personal preference to include it given the current gap in the literature regarding the regulation and function of VSP-II genes.

5) Line 246: “aerB is part of a much longer transcript (extending >1 kb upstream of the start codon).” Did you test for any potential upstream or cryptic ORFs in this upstream region? Could these play a role in the aggregation phenotype or the regulation of aerB?

This is an interesting question. However, since we were able to generate aggregation by overexpressing *aerB* in a strain lacking native VSP-II (Fig. 5A), we conclude that any elements present in this region are dispensable for our aggregative phenotype.

6) Line 212-213: “Neither the transporter nor the che-3 cluster, however, were required for Δzur aggregation (Fig. S1F)." Figure S1F uses gene names while Fig. S3 and the previous text use VC numbers. This makes it difficult to verify which genes are referred to in this sentence. Please edit this sentence or the earlier one to clarify which genes are testing the transporter and the che-3 cluster in Fig. S1F.

We have amended the figure and text.

7) Line 339: missing the word ‘is’, “ the human host is a well-studied…” Corrected.

8) There are a few typos in calling figures. Also, in numerous instances, figures or panels are called out of order. I personally don’t mind this, especially for supplemental figures when it puts similar panels together, but some other readers might find it distracting. If the supplemental figures/panels can be easily rearranged it could be worthwhile. I may have missed others as well, but some examples listed below:

a. Line 283: Fig. S5 appears after S6
b. Line 142: Fig. SF -> Fig. S1F. Also, this appears before S1E
c. Line 290: Fig. 6A -> Fig. 5A; again out of order

Corrected.

Reviewer #3: This is an excellent paper that relies on clever genetic screens, gene expression studies and phenotypic assays to uncover the mechanistic basis for aggregation of V. cholerae lacking Zur, a key regulator of the response to zinc starvation. These multi-faceted studies lead to a highly plausible model to explain the aggregation phenotype. The paper will be of considerable interest to PLOS Genetics readers interested in bacterial genetics and physiology. One short coming of the work, which the authors acknowledge and that should not delay publication, is the absence of experiments to decipher when this mechanism is important for the pathogen. The authors frame the work suggesting that these studies might advance understanding of the predominance of the El Tor biotype, but they don’t deliver on that suggestion.

We thank the reviewer for their feedback on this work.

Major points:
1. Figure 3 should include a MA plot and/or a volcano plot showing all genes and annotated with genes the authors want to highlight; e.g., the authors allude to increased expression of ctxAB and Tcp proteins in the zur deficient background but the reader has no idea of the magnitude of these changes. In Figure 3D, the heat map scale should include numbers.

We have added numbers to the scale in Figure 3D. A Volcano plot is now shown in Supplemental Figure 3, and these data is also provided in Table S2.

2. While it is difficult to discern when VerA/AerB dependent mechanisms confer fitness to the pathogen, there is simple (non-animal) experiment that might provide a clue- qPCR or RNA-seq analysis in El Tor virulence inducing conditions (AKI media).

Since AKI media contains yeast extract (a source of zinc), we anticipate that most zinc starvation genes (including those on VSP-II) will be repressed in this growth medium.

3. Fig 5D is not particularly convincing. Would overexpression of AerB have the opposite effect?

We have included new data (in Figure 5) showing ectopic expression of aerB. In succinate, aerB expression generates a small swarm diameter relative to wild-type. This difference was not observed on maltose plates, consistent with the interpretation that aerB is involved in aerotaxis.

However, we would like to note that in the amended manuscript, we now refer to the chemotactic behavior as oxygen-dependent energy taxis, rather than aerotaxis. We feel that this softened conclusion is more in line with our data.

Minor points:
1. classical biotype is not capitalized.

Corrected

2. Add to discussion recent evidence that VSP-encoded ORFs may be phage defense elements (<https://www.biorxiv.org/content/10.1101/2021.03.31.437871v1> ). Some of these enzymes are actually Zn-dependent - would be nice to see some contextualization/discussion of how these findings relate to each other (if at all). We have added this citation to our discussion section.
3. Line 270 change ‘indicate’ to ‘strongly suggests’ [these data are not definitive]. Changed.
4. Line 290 incorrectly refers to Fig 6A. Corrected.
5. Reference 1 is out of date. Lancet published a new cholera review since then. Changed.
6. Is VerA or AerB under-represented in the in vivo TnSeq publications?

A Tn-Seq study (doi: [10.1371/journal.ppat.1003800](https://doi.org/10.1371/journal.ppat.1003800)) that analyzed fitness in rabbit cecal fluid, rabbit small intestine, and pond water did not detect any significant fitness defects in verA or aerB mutants. We have updated our discussion to include this reference. We note that *znu* transposon mutants exhibited significant fitness defects in the rabbit, suggesting that these environments were sufficient to induce the zinc starvation response.

7. Additional discussion/exploration of the relationship of AerA and AerB would be useful; e.g. are they expressed at the same time?

AerA expression was not altered by Zur and our motility data seem to suggest that native aerA expression on soft agar is sufficient to modulate chemotaxis. We have amended our discussion to make mention of this. To our knowledge, environmental factors that regulate aerA have not been explored in *V. cholerae.*

8. Does classical delta zur aggregate?

The wild-type classical agglutinates in M9 minimal medium, likely due to constitutive tcpA production. We have thus far been unsuccessful in deleting tcp from the classical biotype to test this hypothesis. However, we have now included new data showing that El Tor isolates with non—prototypical VSP-II islands (e.g. C6706 and Haiti) do not aggregate when zur is deleted (Figure 6); However, aggregation can be induced in each of these strains through ectopic *aerB* expression, suggesting that the protein-protein interactions formed by this MCP are not strain-specific.
